# Investigating the Role of A20 in Respiratory Syncytial Virus Immunopathogenesis in a BALB/c Mouse Model

**DOI:** 10.1002/iid3.70337

**Published:** 2026-02-04

**Authors:** Alireza Tahamtan, Mohammad Yasaghi, Saeed Samadizadeh, Hadi Razavi Nikoo, Ahad Yamchi, Vahid Salimi

**Affiliations:** ^1^ Department of Microbiology, Faculty of Medicine Golestan University of Medical Sciences Gorgan Iran; ^2^ Infectious Diseases Research Center Golestan University of Medical Sciences Gorgan Iran; ^3^ Wellcome‐Wolfson Institute for Experimental Medicine Queen's University Belfast Belfast Northern Ireland UK; ^4^ University of Agricultural Sciences and Natural Resources Gorgan Iran; ^5^ Department of Virology Tehran University of Medical Sciences Tehran Iran

**Keywords:** A20, BALB/c, immunopathology, mouse model, respiratory syncytial viruses

## Abstract

**Background:**

Respiratory syncytial virus (RSV) is a leading cause of acute respiratory tract infections in children and the elderly worldwide. RSV pathogenesis is largely driven by exaggerated host immune responses that result in lung injury. In this study, we examined the role of A20 (TNFAIP3), a key regulator of immune signaling, in RSV infection using a BALB/c mouse model.

**Methods:**

Recombinant lentiviruses encoding TNFAIP3 (A20) or A20‐specific shRNA were generated and administered to BALB/c mice. Animals received intravenous lentivectors, challenged intranasally with RSV‐A2, and sacrificed on Day 5 postinfection. A20 expression, cytokine and chemokine levels, lung pathology, and viral load were assessed using real‐time polymerase chain reaction (RT‐PCR), enzyme‐linked immunosorbent assay (ELISA), and histopathological analysis.

**Results:**

RSV infection significantly induced A20 expression in bronchoalveolar (BAL) cells. Lentivector‐mediated modulation of A20 expression produced distinct outcomes: A20 downregulation amplified inflammatory responses, increased immune cell infiltration, and elevated pro‐inflammatory mediator secretion in BAL fluid, leading to aggravated lung pathology. In contrast, A20 upregulation did not markedly alter immune cell recruitment, cytokine production, or histopathological changes following RSV infection.

**Conclusion:**

A20 downregulation exacerbates inflammation and lung injury following RSV infection, highlighting its critical role in immune regulation during the virus infection. Further studies employing targeted molecular delivery systems and human airway organoid models are warranted to evaluate the therapeutic potential of modulating A20 in RSV disease.

**Abbreviations**
BALF: bronchoalveolar lavage fluid
CCL5: chemokine (C‐C motif) ligand 5
cDNA: complementary DNA
COPD: chronic obstructive pulmonary disease
H&E: hematoxylin and eosin
IFN‐β: interferon‐β
IFN‐γ: interferon gamma
IL‐10: interleukin‐10
IRFs: interferon regulatory factors
ISGs: interferon‐stimulated genes
MIP‐1α: macrophage inflammatory protein‐1 alpha
NF‐κB: nuclear factor kappa B
PEG: polyethylene glycol
RIG‐I: retinoic acid‐inducible gene I
RSV: respiratory syncytial virus
RT‐PCR: real‐time reverse transcription polymerase chain reaction
TNFAIP3: tumor necrosis factor α‐induced protein 3
TNF‐α: tumor necrosis factor α

## 1 Introduction

Respiratory syncytial virus (RSV) is the leading cause of bronchiolitis and pneumonia in infants, the elderly, and immunocompromised individuals [1]. Globally, RSV is responsible for more than 33 million acute lower respiratory tract infections, ~3.2 million hospitalizations, and nearly 200,000 deaths among children under 5 years of age [2]. The absence of an effective preventive vaccine, particularly for neonates and young children, contributes to the high burden of RSV infection. The major obstacle to vaccine development lies in the complexity of RSV pathogenesis [3].

RSV‐induced bronchiolitis and pneumonia are strongly associated with an increased risk of asthma, recurrent wheezing, and long‐term pulmonary dysfunction [4]. Moreover, RSV infection frequently coincides with exacerbations of asthma and chronic obstructive pulmonary disease (COPD) [5]. Although the mechanisms underlying RSV‐induced lung disease and its chronic sequelae remain incompletely defined, airway inflammation is thought to play a central role [6]. RSV triggers extensive epithelial injury through excessive inflammatory responses. Cytokines/chemokines, while essential for host defense, are widely regarded as key mediators of RSV immunopathogenesis [7]. Thus, modulating the inflammatory responses through the orchestration of intrinsic signaling represents a promising strategy to mitigate RSV‐associated disease [8].

A20, also known as tumor necrosis factor α‐induced protein 3 (TNFAIP3), is a critical immune regulator that dampens proinflammatory signaling [9]. During RSV infection, airway epithelial cells upregulate proinflammatory and antiviral genes through nuclear factor kappa B (NF‐κB) and interferon regulatory factors (IRFs) activation [8]. NF‐κB, a potent driver of cytokine production in viral infections, can be negatively regulated by A20 [10]. Loss of A20 function results in uncontrolled inflammation and predisposes to autoimmune disorders [11]. Given that RSV pathogenesis is largely driven by dysregulated immune responses, strategies that preserve immune homeostasis are essential to reduce RSV‐related immunopathology. Based on this rationale, the present study investigated the role of A20 in RSV infection and its therapeutic effects using the BALB/c mouse model.

## 2 Material and Methods

### 2.1 Plasmids and Vectors

Codon‐optimized cDNA of the TNFAIP3 gene (A20; 2391 bp), and a 250 bp A20 shRNA were synthesized and cloned into separate pCDH‐CMV‐MCS‐EF1α‐GreenPuro plasmids (Biomatik, Canada). An empty pCDH‐CMV‐MCS‐EF1α‐GreenPuro plasmid served as the control. Plasmids were validated by restriction enzyme digestion and sequencing (data not shown), transformed into *Escherichia coli* TOP10 cells, and purified using the NucleoBond Xtra Maxi Kit (MN, Germany) according to the manufacturer's instructions. Transfection into HEK293 cells confirmed GFP expression.

Recombinant pseudotyped lentiviruses expressing A20, and A20 shRNA, or empty vector were generated by cotransfecting HEK293T cells with pCDH‐CMV‐MCS‐EF1α‐GreenPuro‐TNFAIP3 or shRNA constructs (270 μg), pMDL (gag/pol; 176 μg), pVSVG (95 μg), and pREV (68 μg) using Lipofectamine Plus (Invitrogen, USA). Medium was replaced after 4 h, and supernatants were collected at 48 and 72 h, filtered (0.45 µm), and concentrated by ultracentrifugation. Viral titers were determined by transducing unit (TU) calculation [12]. Vector functionality was confirmed by GFP synthesis (data not shown).

### 2.2 Animal Study

Female BALB/c mice (5–7 weeks old, 15–18 g; 6/group) were obtained from the Pasteur Institute, Karaj, Iran. Animals were acclimatized for 1 week prior to experimentation and housed individually with free access to food and water under controlled conditions (IR.NIMAD.REC.1398.220).

Mice were randomly assigned into different groups (PBS‐PBS, shRNA‐PBS, PBS‐A20, PBS‐RSV, shRNA‐RSV, RSV‐A20, and RSV‐Lenti). The time schedule and experimental purpose are summarized in Supporting Information S2: Table 1. To assess the impact of A20 in the immunopathogenesis of RSV, mice received intravenous lentivectors expressing A20 shRNA (5 × 10^8^ TU/mouse), followed by intranasal RSV challenge (5 × 10^6^ PFU/mouse) the next day. For therapeutic experiments, mice were challenged with RSV (5 × 10^6^ PFU/mouse) and subsequently treated with intravenous A20‐expressing (2 × 10^8^ TU/mouse) 24 h later.

Body weight was monitored daily. Mice were sacrificed on Day 5 postinfection (peak viral load and immune cell influx), and bronchoalveolar lavage fluid (BALF) and lungs were collected for analysis. RSV strain A2 (gift from Professor Louis Bont) was propagated in HEp‐2 cells and concentrated with polyethylene glycol (PEG) [13]. Virus and vectors were resuspended in PBS; controls received a similar volume of PBS or titration of vectors.

### 2.3 A20 Expression

Total RNA was extracted from BAL cells using RNX reagent, and cDNA was synthesized with a High‐Capacity cDNA Reverse Transcription Kit (SinaClone). A20 expression was quantified by real‐time RT‐PCR (ABI PRISM 7900; Applied Biosystems) using SYBR Premix ExTap II (SinaClone). Relative expression was calculated using the 2^−ΔΔCt^ method, normalized to β‐actin. Cycling conditions: 95°C for 30 s, followed by 40 cycles of 95°C for 5 s and 60°C for 1 min. Primer sequences: A20: 5′‐CTGCAAGAACATCCTGGCCT‐3′ (F), 5′‐TGCAGTAGCCGTTGCACT‐3′ (R), and β‐actin: 5′‐GCTCTGGCTCCTAGCACCAT‐3′ (F), 5′‐GCCACCGATCCACACAGAGT‐3′ (R).

### 2.4 Experimental Analysis

BALF was collected on Day 5 postinfection as previously described [13]. Briefly, lungs were lavaged with ice‐cold PBS, centrifuged, and resuspended in 200 μL PBS. Cell counts were performed using Neubauer chambers, and differential leukocyte counts were obtained from Giemsa‐stained smears (1 × 10^5^ cells/200 μL).

Cytokines (MIP‐1α, TNF‐α, IFN‐γ, IL‐10) were quantified by ELISA kits according to the manufacturer's guidelines (Pepro‐Tech, USA). The concentrations of cytokine on BALF supernatants were determined through interpolation from the standard curve, with sensitivity thresholds for MIP‐1α, TNF‐α, IFN‐γ, and IL‐10 set at 4, 16, 16, and 39 pg mL^−1^, respectively.

Lungs were harvested 5 days postinfection [13], followed by overnight fixation in 10% phosphate‐buffered formalin. Subsequently, whole lung samples were embedded in paraffin. Tissue sections (5 μm) were prepared on silane‐coated glass slides and stained with hematoxylin and eosin (H&E). The slides were evaluated using a light microscope, and lung pathology, peribronchial, and perivascular infiltration in the lungs were scored by an experienced clinical pathologist using standard criteria [14, 15]. Group comparisons were conducted based on the average sum of each recorded score.

Viral nucleic acids were isolated from BALF supernatants using a commercially available kit (Roje, Iran). RT‐PCR was conducted on an ABI PRISM 7300 sequence‐detection system (Applied Biosystems, USA) using TaqMan PCR Master Mix (Pishtazteb, Iran). A standard curve was established using predetermined virus concentrations, and the outcomes were quantified in terms of copy numbers per milliliter, as previously outlined [13].

### 2.5 Statistical Analysis

Data were analyzed using GraphPad Prism v6.0 (GraphPad Software, San Diego, CA, USA). Results are expressed as mean ± SEM. Differences between groups were assessed using unpaired Student's *t*‐tests, with statistical significance set at *p *< 0.05. Data quality control and outlier detection are shown in Supporting Information S1: Supplementary Methods.

## 3 Results

**Figure 1:**
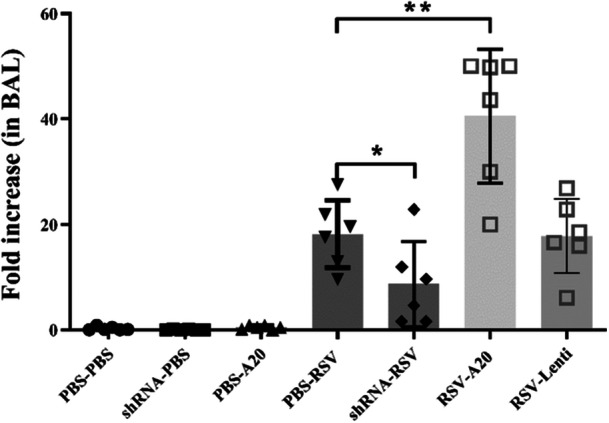
Effect of RSV infection and lentivector administration on A20 expression. Relative A20 expression was assessed in BAL cells of mice 5 days postinfection, using specific primers for the A20 gene and normalized to the housekeeping gene β‐actin. Mice were treated and infected according to different groups (PBS‐PBS, shRNA‐PBS, PBS‐A20, PBS‐RSV, shRNA‐RSV, RSV‐A20, and RSV‐Lenti) as described in Section Methods, 2. Individual data points from each animal (*n *= 6/group) are shown overlaid on the bar graphs. Results are presented as mean ± SEM of 6 animals/group (*p = 0.04, **p = 0.03, and *p* = 0.003; PBS‐RSV vs. uninfected groups).

To assess the impact of primary RSV infection on A20 expression, we quantified A20 levels in BAL cells 5 days postinfection. RSV‐infected mice exhibited a marked increase in A20 expression compared with uninfected controls (*p *< 0.0001). Lentivector‐mediated A20 downregulation significantly reduced A20 expression (*p *= 0.04), whereas A20 overexpression significantly enhanced its expression following RSV infection (*p *= 0.003) (Figure 1).

**Figure 2:**
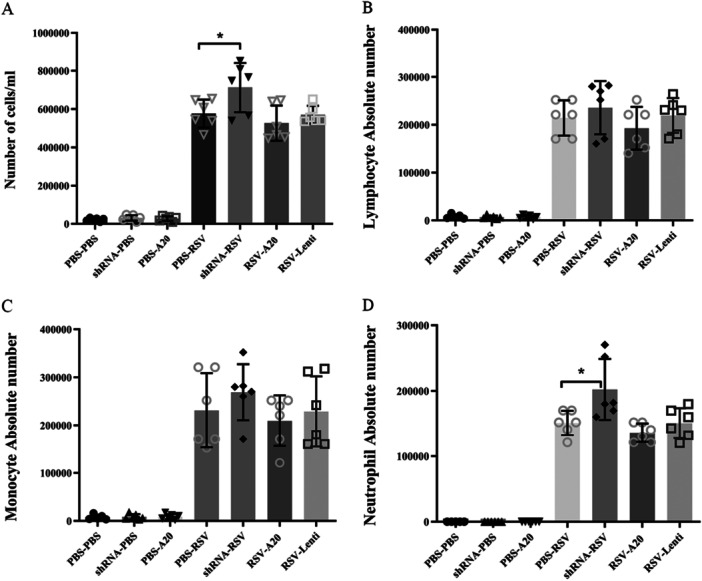
Effect of A20 modulation on immune cell influx following RSV infection. Mice were treated and infected according to different groups (PBS‐PBS, shRNA‐PBS, PBS‐A20, PBS‐RSV, shRNA‐RSV, RSV‐A20, and RSV‐Lenti) as described in Section Methods, 2. Total BAL cell counts (A), lymphocytes (B), monocytes (C), and neutrophils (D) were quantified on Day 5 postinfection. Individual data points from each mouse (*n *= 6/group) are shown overlaid on the bar graphs. Results are expressed as mean ± SEM of 6 animals/group (*p < 0.05).

Primary RSV infection triggers a robust inflammatory response characterized by leukocyte influx into the pulmonary airways as shown in the PBS‐RSV group (Figure 2). BALF analysis revealed that A20 downregulation significantly increased total infiltrating cell counts (575,000 → 712,000; *p*= 0.04). Morphological assessment showed elevated lymphocyte, monocyte, and neutrophil numbers, with neutrophils being significantly increased (*p *= 0.03). A20 overexpression reduced immune cells infiltration, though these decreases did not reach statistical significance.

**Figure 3:**
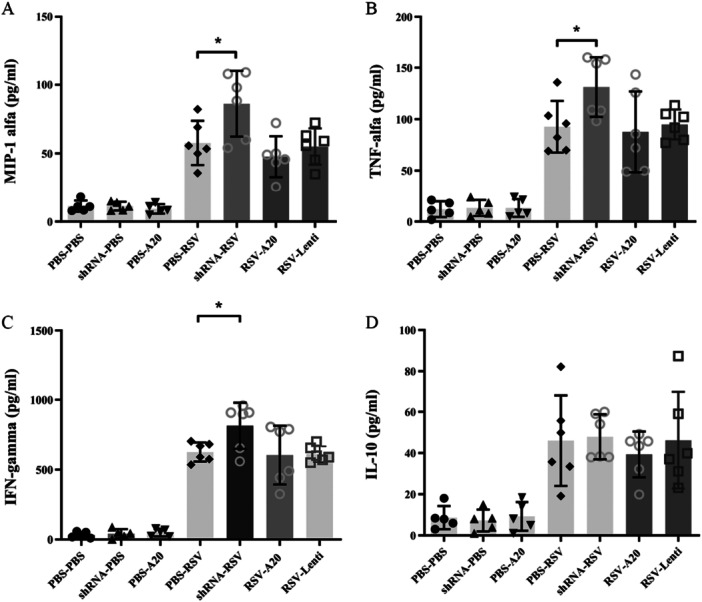
Effect of A20 modulation on cytokine production following RSV infection. Mice were treated and infected according to different groups (PBS‐PBS, shRNA‐PBS, PBS‐A20, PBS‐RSV, shRNA‐RSV, RSV‐A20, and RSV‐Lenti) as described in Section Methods, 2. Levels of MIP‐1α (A), TNF‐α (B), IFN‐γ (C), and IL‐10 (D) were measured in BAL supernatants on Day 5 postinfection. Individual data points from each mouse (*n *= 6/group) are shown overlaid on the bar graphs. Results are expressed as mean ± SEM of 6 animals/group (*p < 0.05).

Cytokine profiling demonstrated that A20 downregulation augmented pro‐inflammatory mediator production, including MIP‐1α (*p *= 0.03), TNF‐α (*p *= 0.03), and IFN‐γ (*p *= 0.02) (Figure 3). Conversely, A20 overexpression reduced cytokine levels, though these decreases did not reach statistical significance.

**Figure 4:**
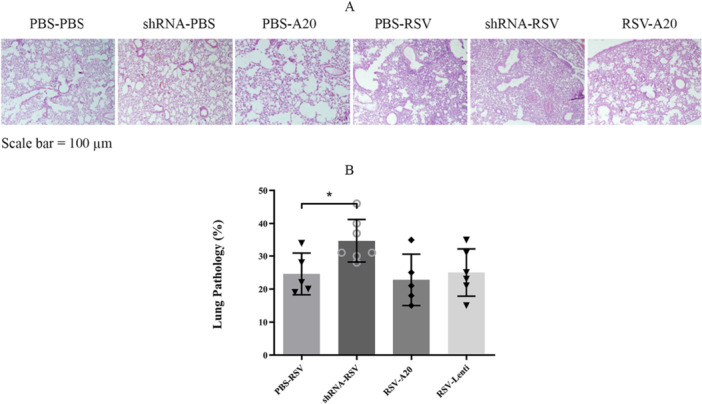
Effect of A20 modulation on lung pathology following RSV infection. Mice were treated and infected according to different groups (PBS‐PBS, shRNA‐PBS, PBS‐A20, PBS‐RSV, shRNA‐RSV, RSV‐A20, and RSV‐Lenti) as described in Section Methods, 2. Lung pathology was assessed on Day 5 postinfection. Representative hematoxylin and eosin (H&E)‐stained lung sections (A) were analyzed and scored (B). Individual data points from each mouse (*n *= 6/group) are shown overlaid on the bar graphs. Results are expressed as mean ± SEM of 6 animals/group (*p < 0.05). Scale bar for all microscopic images = 100 µm.

Histopathological evaluation revealed that RSV infection induced peribronchial and perivascular inflammation, with a pathology score of 24% in PBS‐RSV mice. A20 downregulation exacerbated lung pathology (34%; *p *= 0.02) (Figure 4). A20 overexpression attenuated tissue damage, but the reduction was not statistically significant.

**Figure 5:**
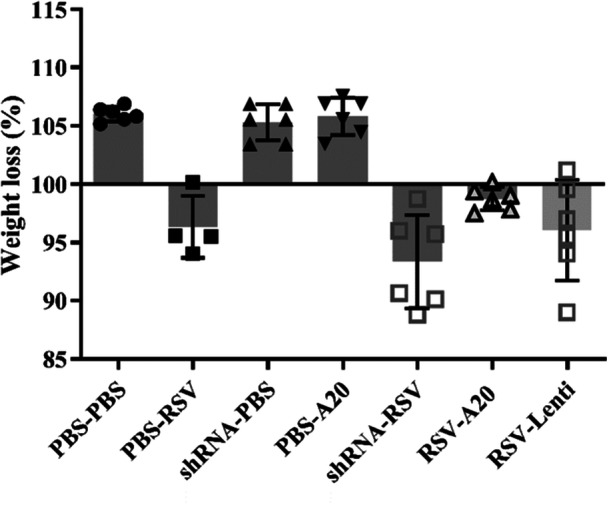
Effect of A20 modulation on body weight loss following RSV infection. Mice were treated and infected according to different groups (PBS‐PBS, shRNA‐PBS, PBS‐A20, PBS‐RSV, shRNA‐RSV, RSV‐A20, and RSV‐Lenti) as described in Section Methods, 2. Changes in body weight were monitored 5 days postinfection. Individual data points from each mouse (*n* = 6/group) are shown overlaid on the bar graphs. Results are expressed as mean ± SEM of 6 animals/group.

**Figure 6:**
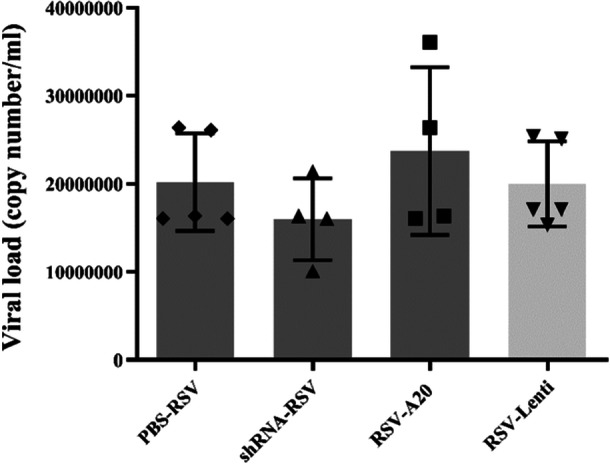
Effect of A20 modulation on viral load following RSV infection. Mice were treated and infected according to different groups (PBS‐PBS, shRNA‐PBS, PBS‐A20, PBS‐RSV, shRNA‐RSV, RSV‐A20, and RSV‐Lenti) as described in Section Methods, 2. RSV copy numbers in BAL supernatants were quantified by real‐time PCR on Day 5 postinfection. Individual data points from each mouse (*n *= 6/group) are shown overlaid on the bar graphs. Results are expressed as mean ± SEM of 6 animals/group.

Indicators of disease severity further supported these findings. A20 downregulation tended to worsen RSV‐associated weight loss, whereas A20 overexpression partially mitigated it (Figure 5). Viral load analysis showed a nonsignificant trend toward reduction with A20 downregulation and elevation with A20 overexpression (Figure 6).

Importantly, in the absence of RSV infection, administration of shRNA or A20‐inducing lentivectors did not affect A20 expression, leukocyte recruitment, cytokine secretion, lung pathology, or body weight, underscoring the RSV‐specific nature of the observed effects. Furthermore, treatment with the empty vector (RSV‐Lenti group) resulted in changes comparable to those in the PBS‐RSV group, confirming that the observed outcomes were specific to A20‐ or shRNA‐encoding lentivectors.

## 4 Discussion

In this study, A20 expression was downregulated using a lentivector carrying A20‐targeting shRNA to investigate its role in RSV‐induced pathogenesis. Downregulation of A20 increased immune cell infiltration and elevated inflammatory cytokines and chemokines in BALF, consistent with RSV‐associated immunopathogenesis. Conversely, mice injected with an A20‐expressing lentivector exhibited reduced lung pathology, characterized by fewer infiltrating immune cells and diminished inflammatory mediators, although these effects did not reach statistical significance. shRNA administration prior to RSV infection was designed to evaluate the role of endogenous A20 during the initial stages of viral recognition and immune activation. A20 lentivirus administration after RSV infection was intended as a therapeutic intervention model.

RSV pathogenesis is driven by immune‐mediated mechanisms, necessitating investigation of regulatory molecules such as A20. A20 functions as a negative regulator of NF‐κB signaling by modulating ubiquitin‐dependent cascades, inhibiting NF‐κB and IRF3/7 transcriptional activity, and thereby limiting inflammation [16]. Previous studies have implicated A20 in viral infections including RSV (in vitro study), influenza, and rotavirus, as well as in nonviral inflammatory and autoimmune diseases [17, 18, 19, 20]. During RSV infection, recognition of viral RNA by RIG‐I activates NF‐κB and IRF3, leading to the production of interferon‐β (IFN‐β), IFN‐λ, interferon‐stimulated genes (ISGs), CCL5, and other inflammatory mediators that drive mucus hyper secretion and leukocyte infiltration, culminating in lung pathology [21]. A20 restricts this antiviral signaling by removing K63‐linked polyubiquitin chains from TBK1/IKKi kinases, thereby suppressing IRF and IFN responses [16, 18, 22, 23]. Additionally, A20 competes with ubiquitin‐binding proteins such as NEMO and interacts with TAX1BP1 and ABIN1, impairing downstream NF‐κB and IRF3 activation [22, 24, 25].

In our animal model, RSV infection enhanced immune cell infiltration in BALF and increased levels of MIP‐1α, TNF‐α, IFN‐γ, and IL‐10, accompanied by lung pathology and weight loss. Consistent with Salinas et al. [15], A20 downregulation amplified immune responses, with elevated MIP‐1α, TNF‐α, and IFN‐γ compared to PBS‐RSV controls. This underscores A20's modulatory role in innate immunity against RSV. Increased neutrophil infiltration further exacerbated pathology and weight loss. Similar findings were reported by Maelfait and colleagues in influenza‐infected mice [18], although in that context heightened inflammation improved viral clearance and reduced disease severity. Given that BALB/c mice are semi‐permissive to RSV, our viral load data may not fully capture host–virus dynamics, leaving unresolved whether RSV disease severity reflects viral replication, excessive inflammation, or both. Notably, A20 can also activate the noncanonical NF‐κB pathway through noncatalytic mechanisms [26], though this effect was not detected in our time‐limited experiments. Prior work showed transient increases in inflammatory mediators at 24 hpi in A20‐deficient A549 cells, which subsided by 48 hpi, possibly due to RSV‐mediated activation of the noncanonical pathway or involvement of partner molecules [17, 27].

Although A20 upregulation led to slightly reduced immune cell infiltration and lower levels of inflammatory mediators, these changes did not effect on disease mitigation. However, this limited impact does not preclude A20's therapeutic potential. Several technical and biological factors may underlie these neutral findings. Notably, gene upregulation studies often encounter greater challenges than knockdown approaches, including risks of off‐target effects, nonphysiological expression levels, and inefficiencies in transfection or viral vector delivery. In this study, intravenous administration may have further constrained efficacy, as it is likely less effective than intratracheal instillation for targeting airway epithelial cells. Therefore, while A20 overexpression showed a protective trend, more refined delivery strategies and optimized study designs are essential to fully assess its therapeutic value.

Investigating the role of A20 in RSV pathogenesis presents notable limitations, particularly concerning the choice of study models and the efficiency of current experimental techniques. To address these challenges, we recommend employing well‐differentiated primary airway epithelial cells or airway organoid models of RSV infection, as they more accurately recapitulate the physiological context of host factors and the mechanisms underlying viral infection [28, 29]. Furthermore, incorporating different stages of disease progression into experimental designs is essential for capturing the dynamic nature of host–virus interactions, which may influence either the exacerbation or resolution of pathogenesis.

With respect to A20, it is critical to explore functionally related molecules within the same or parallel signaling pathways to determine whether compensatory mechanisms exist in response to its dysregulation. This includes examining A20's interactions with partner proteins across various signaling cascades, as these interactions may yield divergent functional outcomes depending on the molecular context [30, 31, 32, 33]. Additionally, viral proteins such as NS1 from influenza and RSV have been shown to subvert host cellular machinery to facilitate viral replication and dissemination through mechanisms like immune evasion and apoptosis modulation [34, 35]. Therefore, elucidating the impact of RSV infection on A20 expression and function could significantly advance our understanding of its role in viral pathogenesis and inform potential therapeutic strategies.

Several limitations of this study warrant consideration. First, intravenous lentivector delivery likely resulted in suboptimal transduction of lung epithelial cells, thereby limiting the efficacy of A20 overexpression; intratracheal administration may offer superior targeting. Second, the lack of statistical significance in the A20 overexpression cohort highlights the need for more efficient delivery systems or cell‐specific targeting strategies. Third, the use of a scrambled shRNA control would strengthen the interpretation of A20 knockdown effects by ruling out off‐target contributions. Fourth, while the BALB/c mouse model is widely used, its semi‐permissiveness to RSV infection limits its translational relevance to human disease. Finally, the analysis was confined to a single time point (5 days postinfection), which may have overlooked critical temporal dynamics in disease progression and immune response.

## 5 Conclusions

This study is the first to explore A20's role in RSV infection using an animal model. It shows that A20 downregulation worsens inflammation and lung damage, highlighting its regulatory importance. However, A20 overexpression did not yield statistically significant benefits, leaving its therapeutic potential uncertain. Future research should focus on improved delivery methods, the use of human‐relevant models like airway organoids, and time‐course analyses. Investigating A20's molecular partners and RSV's potential to disrupt A20 function may uncover new therapeutic insights.

## Author Contributions

**Alireza Tahamtan:** conceptualization, methodology, project administration, supervision, validation, visualization, writing – review and editing. **Mohammad Yasaghi:** data curation, formal analysis, investigation, writing – original draft. **Saeed Samadizadeh:** data curation, formal analysis, investigation. **Hadi Razavi Nikoo:** validation, writing – review and editing. **Ahad Yamchi:** validation, writing – review and editing. **Vahid Salimi:** validation, writing – review and editing. All authors have read and approved the final version of the manuscript, had full access to all of the data in this study and take complete responsibility for the integrity of the data and the accuracy of the data analysis.

## Ethics Statement

The experiment was in accordance with the standard protocols approved by the Ethics Committee of the Golestan University of Medical Sciences (IR.NIMAD.REC.1398.220).

## Conflicts of Interest

The authors declare no conflicts of interest.

## Supporting information


**Supplementary Table 1:** The time schedule and experimental purpose.

Supplementary Methods.docx.

## Data Availability

The authors confirm that the data supporting the findings of this study are available within the article.
