# Supplementary material for: Investigating the Role of A20 in Respiratory Syncytial Virus Immunopathogenesis in a BALB/c Mouse Model
Source: Immun Inflamm Dis. 2026 Feb 4;14(2):e70337. doi: 10.1002/iid3.70337 (PMC12872964; doi:10.1002/iid3.70337)
Supplement: Supplementary file 1 — Supplementary Table 1: The time schedule and experimental purpose. [file IID3-14-e70337-s002.docx]

**Supplementary Methods: Data quality control and outlier detection**

Prior to statistical analysis, all individual data points were screened for potential outliers. Outlier detection was conducted using Grubb’s test (α = 0.05), which identifies values that deviate significantly from group distributions and are most likely attributable to technical or measurement error rather than biological variation.

In the majority of experimental groups (Figures 1–4), no outliers were detected, and all animals (n = 6 per group) were included in the analyses. In contrast, one data point from the shRNA-RSV group (Figure 6) and one data point from the shRNA-RSV and RSV-A20 groups (Figure 5) were identified as extreme outliers and excluded.

This conservative quality-control strategy preserves the integrity of statistical conclusions while maintaining transparency regarding sample sizes. Importantly, the exclusion of these outliers is consistent with established biostatistical practice and does not alter the biological interpretation of the findings.
