# Supplementary material for: Investigating the Role of A20 in Respiratory Syncytial Virus Immunopathogenesis in a BALB/c Mouse Model
Source: Immun Inflamm Dis. 2026 Feb 4;14(2):e70337. doi: 10.1002/iid3.70337 (PMC12872964; doi:10.1002/iid3.70337)
Supplement: Supplementary file 2 — Supplementary Methods.docx. [file IID3-14-e70337-s001.docx]

**Supplementary Table 1:** The time schedule and experimental purpose

| **Experimental Groups** | **Day 0** | **Day 1** | **Day 5** | **Purpose** |
| --- | --- | --- | --- | --- |
| **PBS-PBS** | PBS (IV) | PBS (IN) | Sacrifice & Analysis | Vehicle control (no treatment) |
| **shRNA-PBS** | A20 shRNA lentivector (5 × 10⁸ TU/mouse, IV) | PBS (IN) | Sacrifice & Analysis | shRNA control (gene knockdown with no infection) |
| **PBS-A20** | PBS (IN) | A20 (IV) | Sacrifice & Analysis | A20 overexpression control with no infection |
| **PBS-RSV** | PBS (IV) | RSV-A2 (5 × 10⁶ PFU/mouse, IN) | Sacrifice & Analysis | RSV infection baseline |
| **shRNA-RSV** | A20 shRNA lentivector (5 × 10⁸ TU/mouse, IV) | RSV-A2 (5 × 10⁶ PFU/mouse, IN) | Sacrifice & Analysis | *A20 downregulation effects on RSV infection (A20 downregulation before infection) |
| **RSV-A20** | RSV-A2 (5 × 10⁶ PFU/mouse, IN) | A20-expressing lentivector (2 × 10⁸ TU/mouse, IV) | Sacrifice & Analysis | *A20 overexpression effects on RSV infection (therapeutic, post-infection) |
| **RSV-Lenti** | RSV-A2 (5 × 10⁶ PFU/mouse, IN) | Empty vector (2 × 10⁸ TU/mouse, IV) | Sacrifice & Analysis | Empty vector control (post-infection, delivery toxicity control) |

* To assess the impact of A20 in the immunopathogenesis of RSV, mice received intravenous lentivectors expressing A20 shRNA (5 × 10⁸ TU/mouse), followed by intranasal RSV challenge (5 × 10⁶ PFU/mouse) the next day. For therapeutic experiments, mice were challenged with RSV (5 × 10⁶ PFU/mouse) and subsequently treated with intravenous A20-expressing (2 × 10⁸ TU/mouse) 24 h later. Virus and vectors were resuspended in PBS; controls received a similar volume of PBS or titration of vectors. IV, intravenous; IN, intranasal; TU, transducing units; PFU, plaque-forming units.
